# Supplementary material for: Local immunoglobulin expression in myositis is associated with interferon gamma signaling and correlates with disease activity
Source: medRxiv. 2025 Jun 4:2025.06.03.25328909. Preprint. [Version 1] doi: 10.1101/2025.06.03.25328909 (PMC12155027; doi:10.1101/2025.06.03.25328909)
Supplement: Supplement 1 [file NIHPP2025.06.03.25328909v1-supplement-1.pdf]

medRxiv preprint doi: https://doi.org/10.1101/2025.06.03.25328909; this version posted June 4, 2025. The copyright holder for this preprint (which was not certified by peer review) is the author/funder, who has granted medRxiv a license to display the preprint in perpetuity. This article is a US Government work. It is not subject to copyright under 17 USC 105 and is also made available for use under a CC0 license.

**Supplementary Figure 1. Spearman correlations between immunoglobulin heavy chain gene expression and markers of disease activity across study groups.** Shown are correlations with type I interferon-inducible genes (ISG15, MX1), type II interferon-inducible genes (GBP2, IFI30), T-cell markers (CD3E, CD4, CD8), macrophage markers (CD14, CD68), muscle differentiation markers (MYH3, MYH8, NCAM1, PAX7), mitochondrial genes (MT-CO1, MT-CO2), and structural mature muscle proteins (ACTA1, MYH1, MYH2).

## Normal muscle biopsy

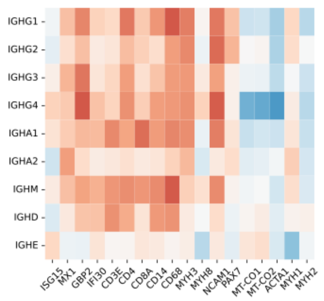


## Anti-Mi2

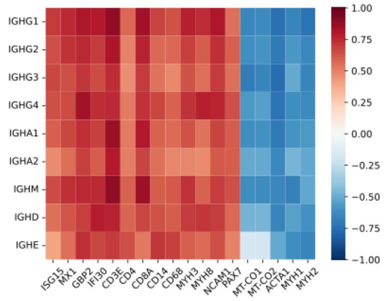


## Anti-TIF1□

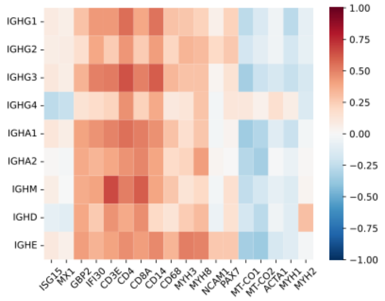


## Anti-NXP2

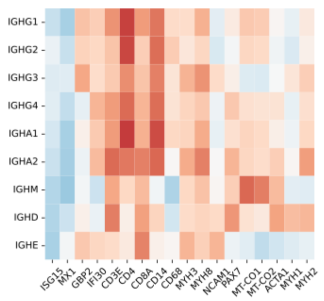


## Anti-MDA5

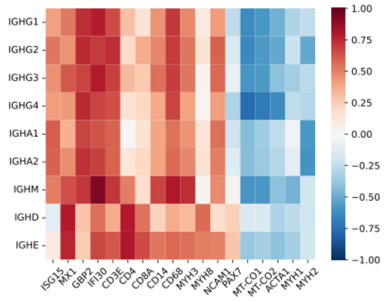


## Anti-Jo1

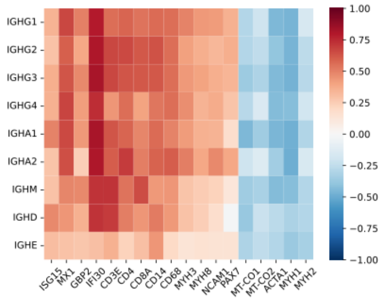


## Anti-HMGCR

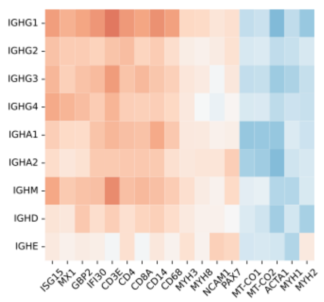


## Anti-SRP

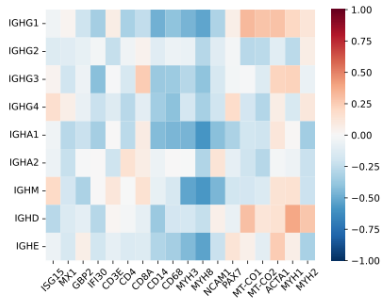


## Inclusion body myositis

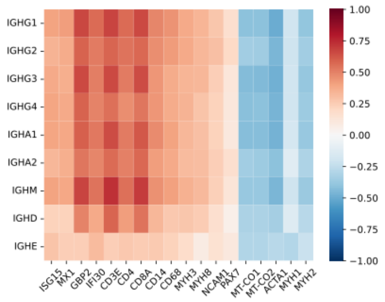

medRxiv preprint doi: https://doi.org/10.1101/2025.06.03.25328909; this version posted June 4, 2025. The copyright holder for this preprint (which was not certified by peer review) is the author/funder, who has granted medRxiv a license to display the preprint in perpetuity.
This article is a US Government work. It is not subject to copyright under 17 USC 105 and is also made available for use under a CC0 license.

**Supplementary Figure 2. Expression of immunoglobulin kappa and lambda light chain constant region genes across disease groups.** Each point represents an individual sample. Expression is presented as trimmed mean of M values (TMM). Normal muscle biopsy: NT; inclusion body myositis: IBM.

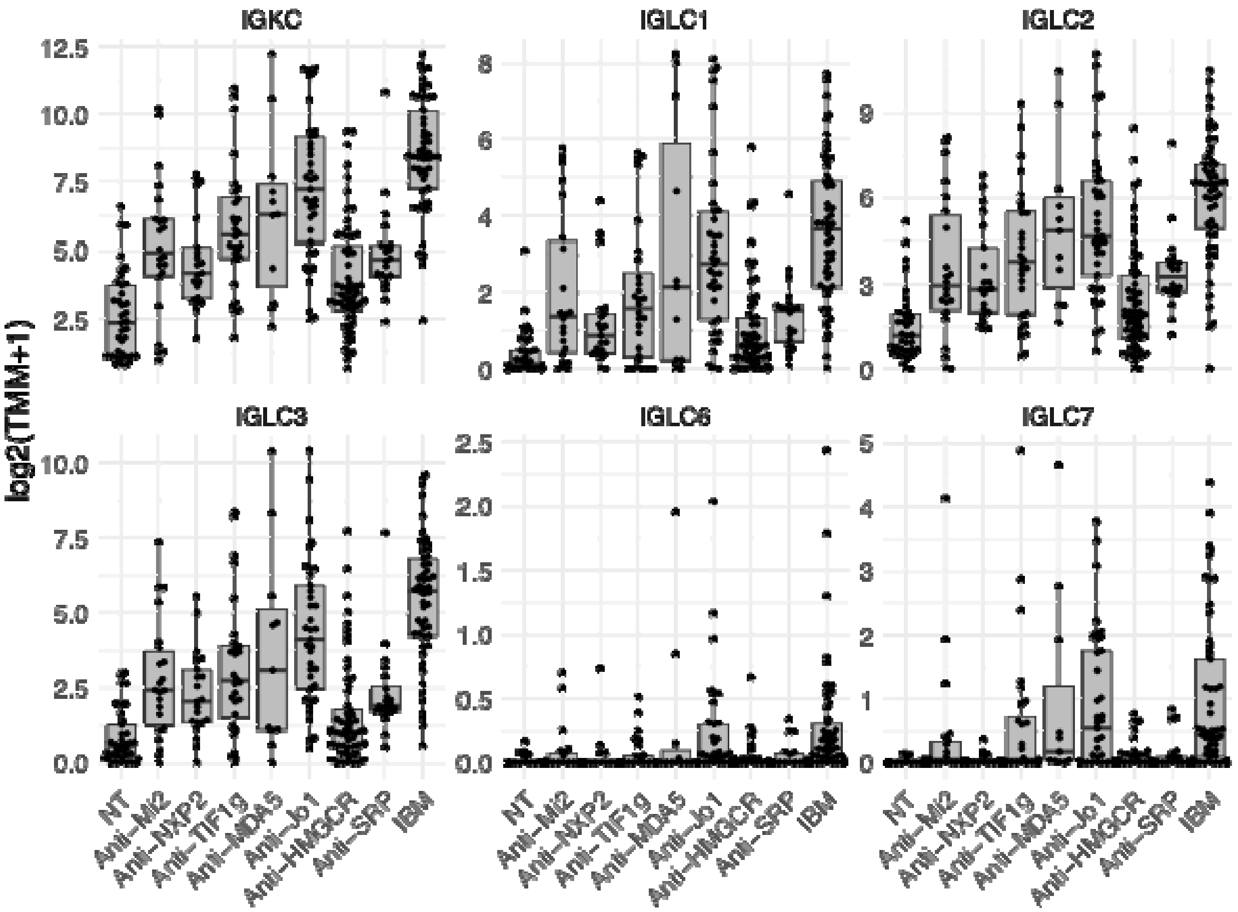

medRxiv preprint doi: https://doi.org/10.1101/2025.06.03.25328909; this version posted June 4, 2025. The copyright holder for this preprint (which was not certified by peer review) is the author/funder, who has granted medRxiv a license to display the preprint in perpetuity.
This article is a US Government work. It is not subject to copyright under 17 USC 105 and is also made available for use under a CC0 license.

**Supplementary Figure 3. Expression of immunoglobulin heavy chain variable region genes across disease groups.** Each point represents an individual sample. Expression is presented as trimmed mean of M values (TMM). Normal muscle biopsy: NT; inclusion body myositis: IBM.

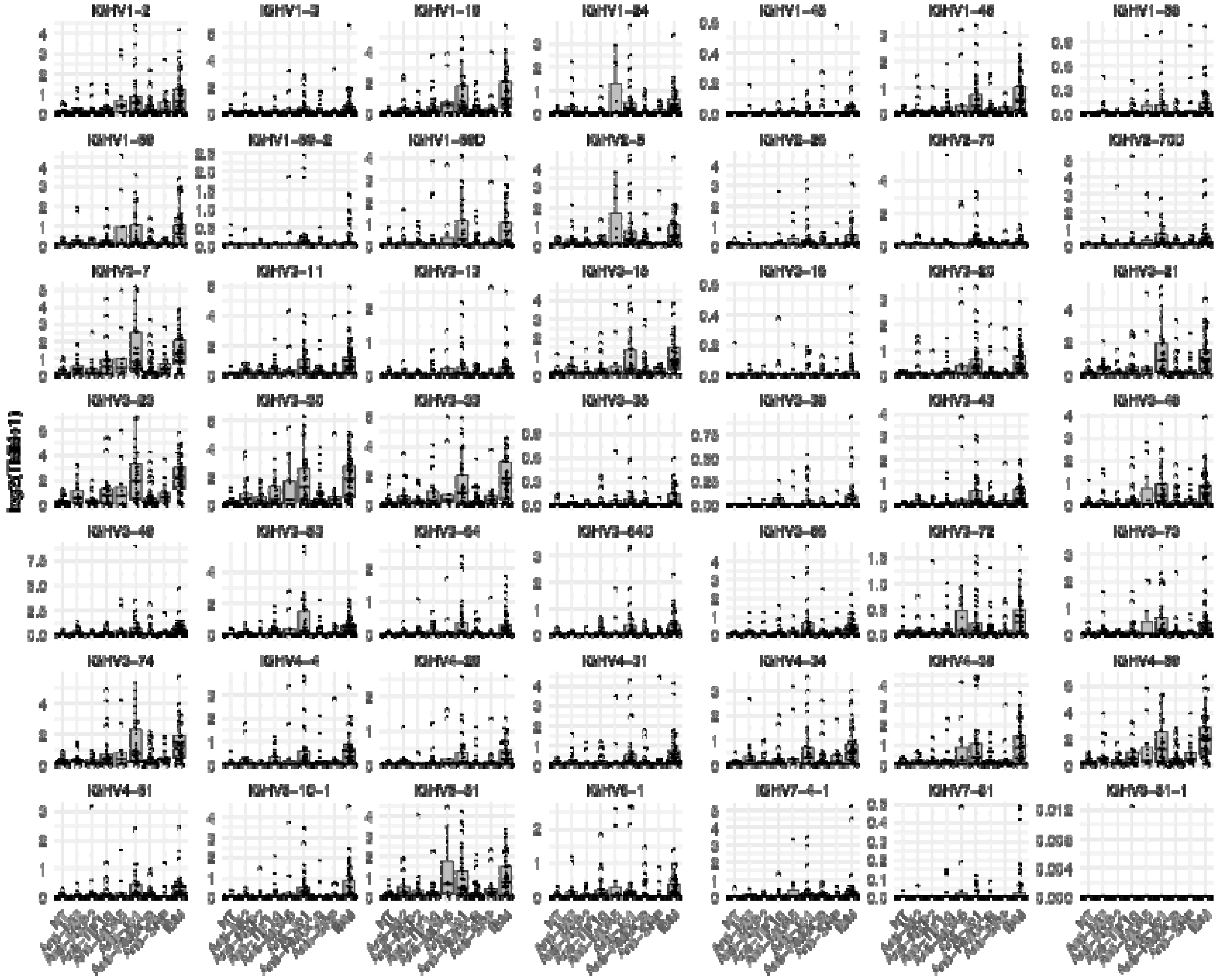

medRxiv preprint doi: https://doi.org/10.1101/2025.06.03.25328909; this version posted June 4, 2025. The copyright holder for this preprint
(which was not certified by peer review) is the author/funder, who has granted medRxiv a license to display the preprint in perpetuity.
This article is a US Government work. It is not subject to copyright under 17 USC 105 and is also made available
for use under a CC0 license.

**Supplementary Figure 4. Expression of immunoglobulin heavy chain joining region genes across disease groups.** Each point represents an individual sample. Expression is presented as trimmed mean of M values (TMM). Normal muscle biopsy: NT; inclusion body myositis: IBM.

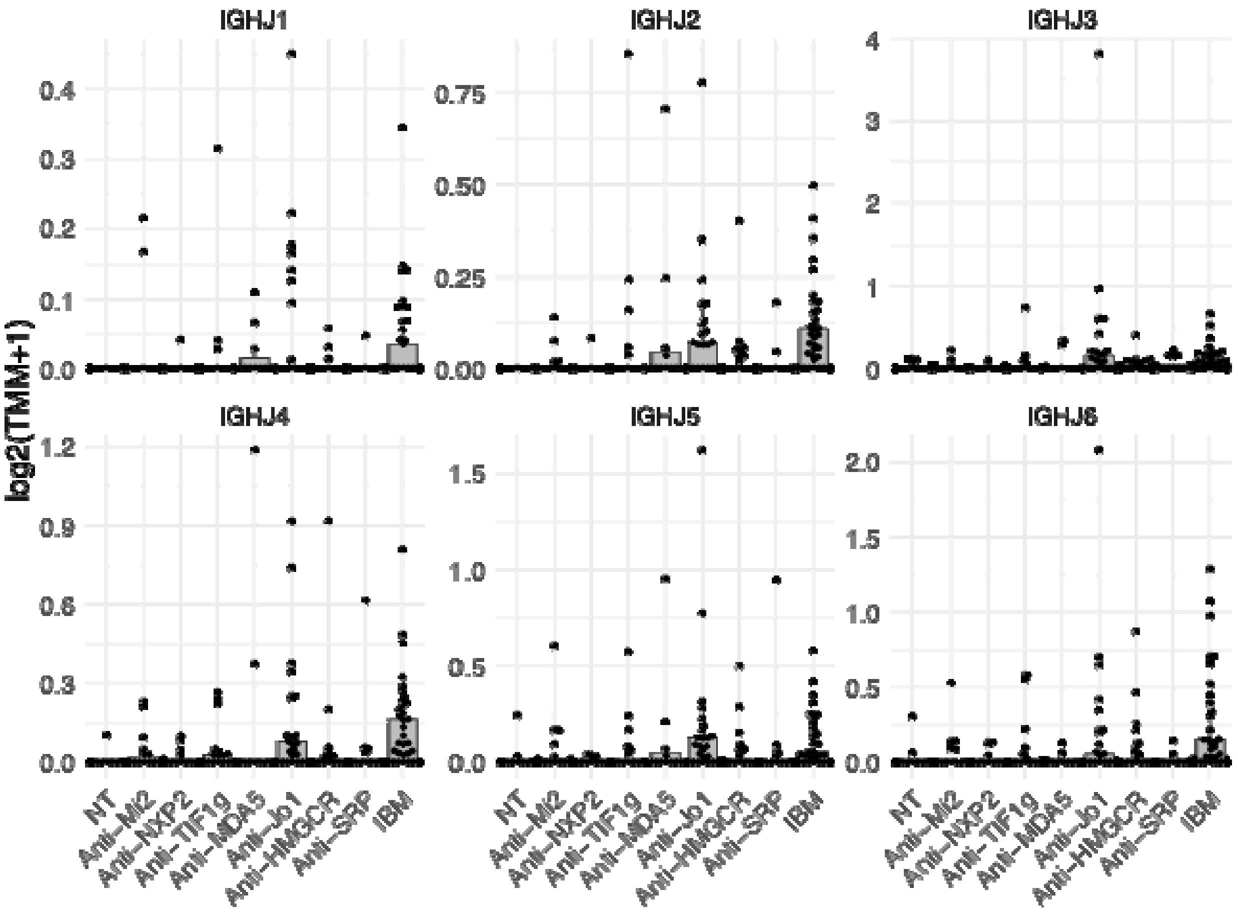

medRxiv preprint doi: https://doi.org/10.1101/2025.06.03.25328909; this version posted June 4, 2025. The copyright holder for this preprint (which was not certified by peer review) is the author/funder, who has granted medRxiv a license to display the preprint in perpetuity.
This article is a US Government work. It is not subject to copyright under 17 USC 105 and is also made available for use under a CC0 license.

**Supplementary Figure 5. Expression of immunoglobulin kappa light chain variable region genes across disease groups.** Each point represents an individual sample. Expression is presented as trimmed mean of M values (TMM). Normal muscle biopsy: NT; inclusion body myositis: IBM.

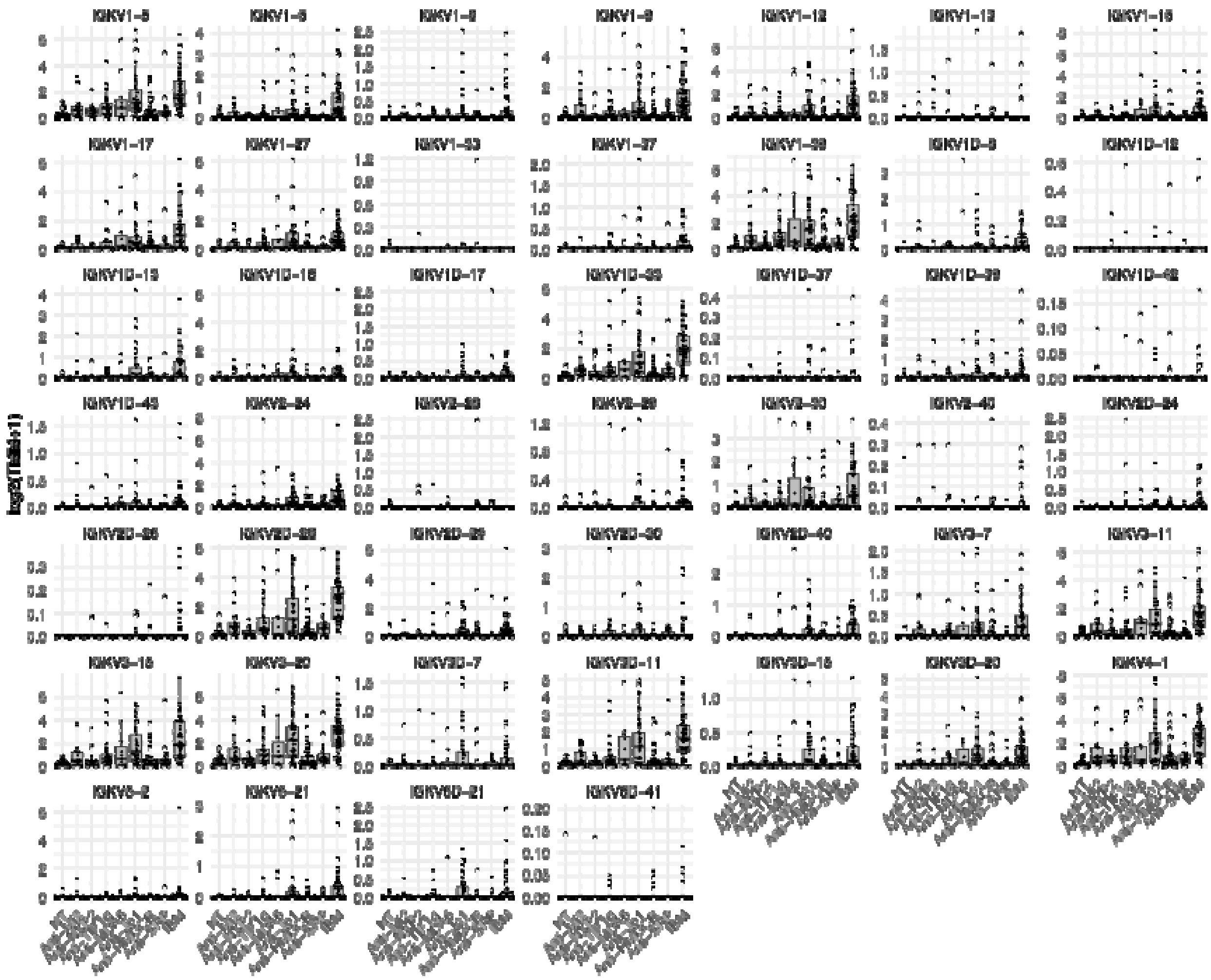

medRxiv preprint doi: https://doi.org/10.1101/2025.06.03.25328909; this version posted June 4, 2025. The copyright holder for this preprint (which was not certified by peer review) is the author/funder, who has granted medRxiv a license to display the preprint in perpetuity. This article is a US Government work. It is not subject to copyright under 17 USC 105 and is also made available for use under a CC0 license.

**Supplementary Figure 6. Expression of immunoglobulin lambda light chain variable region genes across disease groups.** Each point represents an individual sample. Expression is presented as trimmed mean of M values (TMM). Normal muscle biopsy: NT; inclusion body myositis: IBM.

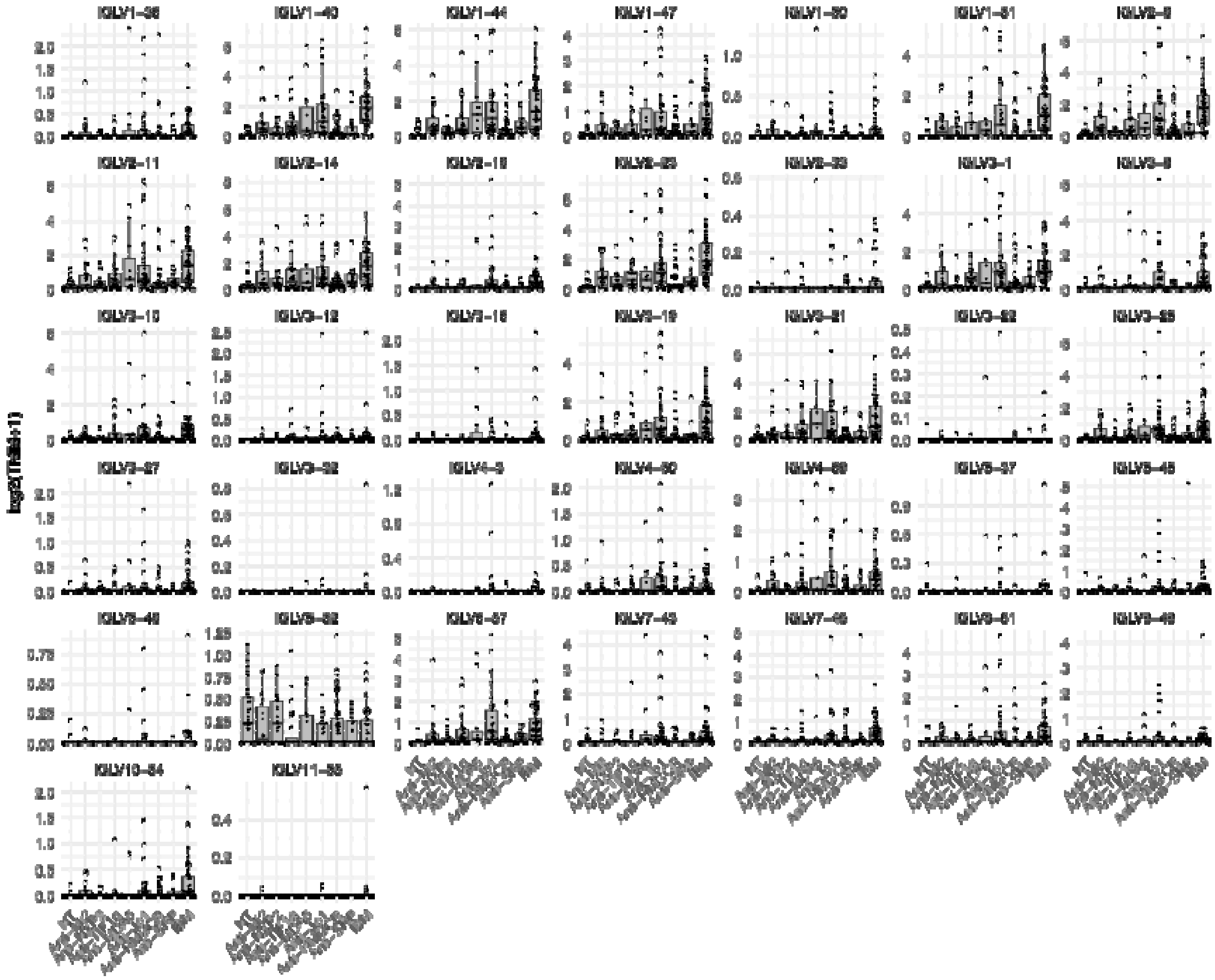

medRxiv preprint doi: https://doi.org/10.1101/2025.06.03.25328909; this version posted June 4, 2025. The copyright holder for this preprint (which was not certified by peer review) is the author/funder, who has granted medRxiv a license to display the preprint in perpetuity.
This article is a US Government work. It is not subject to copyright under 17 USC 105 and is also made available for use under a CC0 license.

# Supplementary Figure 7. Spearman correlation matrix of immunoglobulin genes.

## Heavy chain constant region

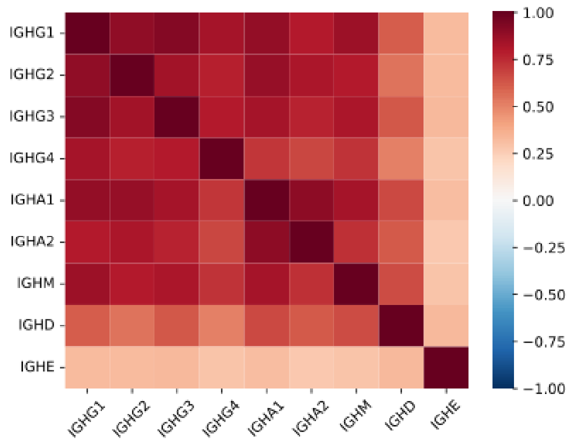


## Kappa/lambda constant regions

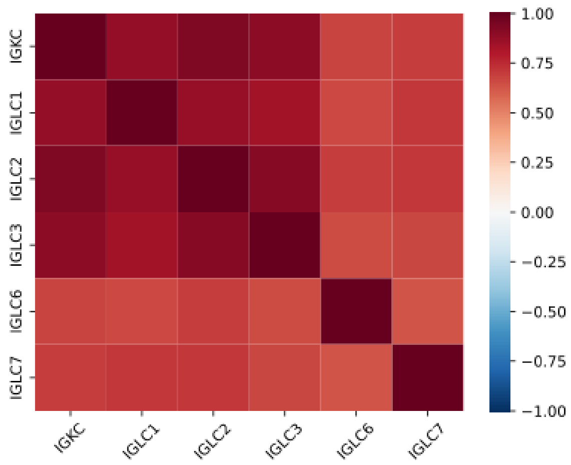


## Heavy chain variable region

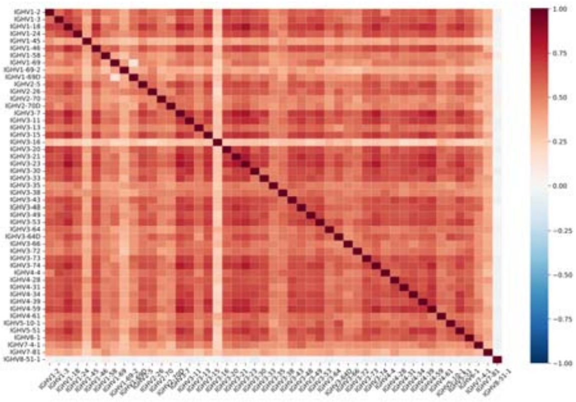

## Heavy chain joining region

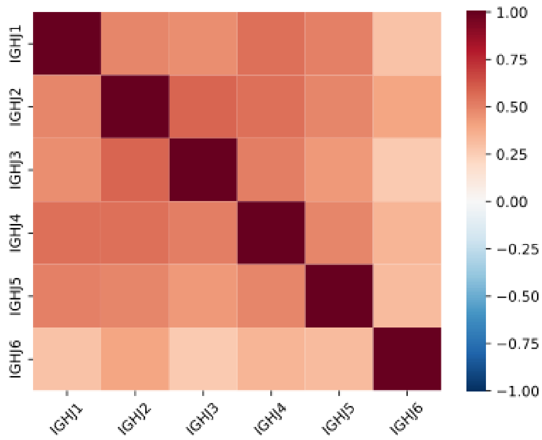


## Kappa chain variable region

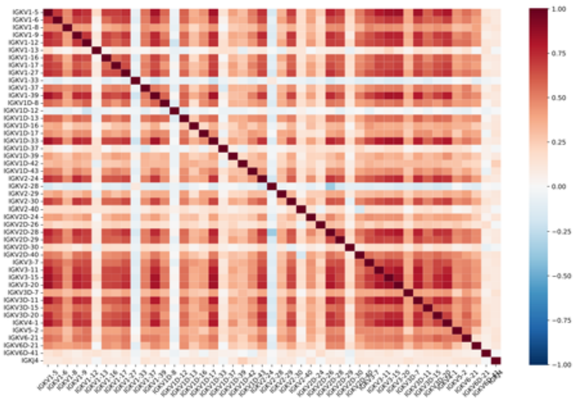

## Lambda chain variable region

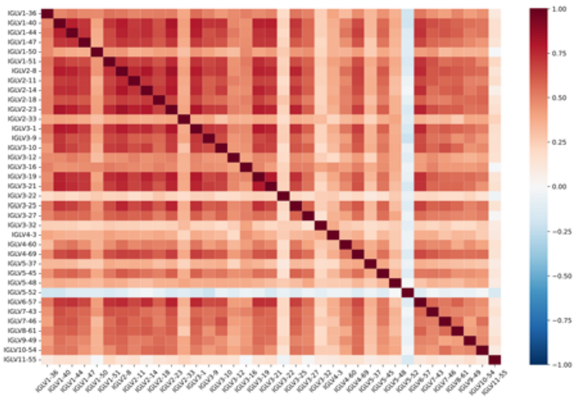

medRxiv preprint doi: https://doi.org/10.1101/2025.06.03.25328909; this version posted June 4, 2025. The copyright holder for this preprint (which was not certified by peer review) is the author/funder, who has granted medRxiv a license to display the preprint in perpetuity. This article is a US Government work. It is not subject to copyright under 17 USC 105 and is also made available for use under a CC0 license.

**Supplementary Figure 8. Spearman correlation between immunoglobulin gene expression and markers of disease activity.** Shown are correlations with type I interferon-inducible genes (ISG15, MX1), type II interferon-inducible genes (GBP2, IFI30), T-cell markers (CD3E, CD4, CD8), macrophage markers (CD14, CD68), muscle differentiation markers (MYH3, MYH8, NCAM1, PAX7), mitochondrial genes (MT-CO1, MT-CO2), and structural mature muscle proteins (ACTA1, MYH1, MYH2).

## Heavy chain constant region

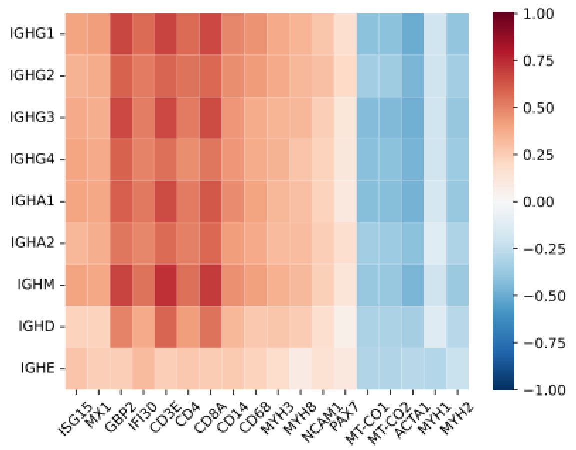


## Kappa/lambda constant regions

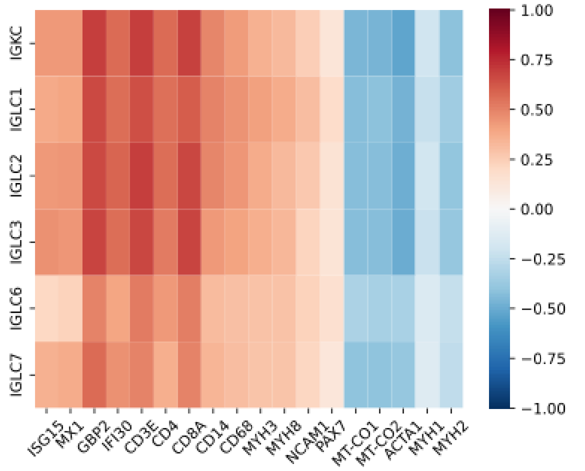


## Heavy chain variable region

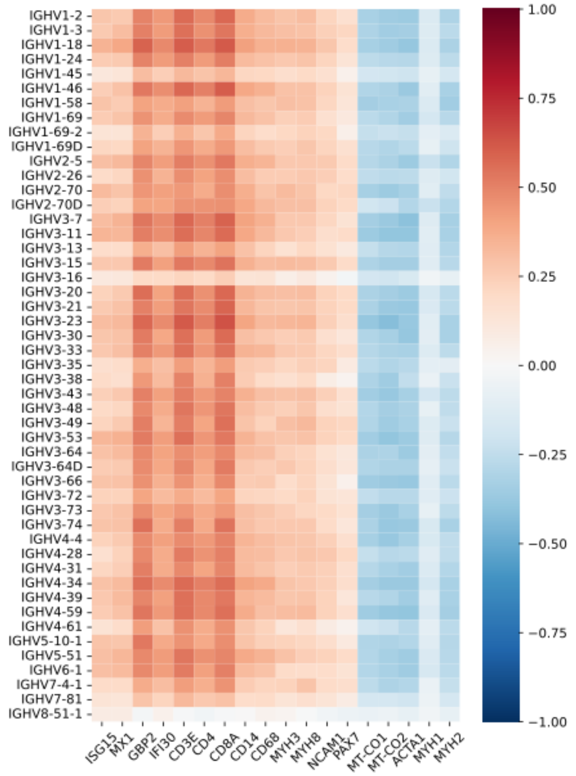


## Heavy chain joining region

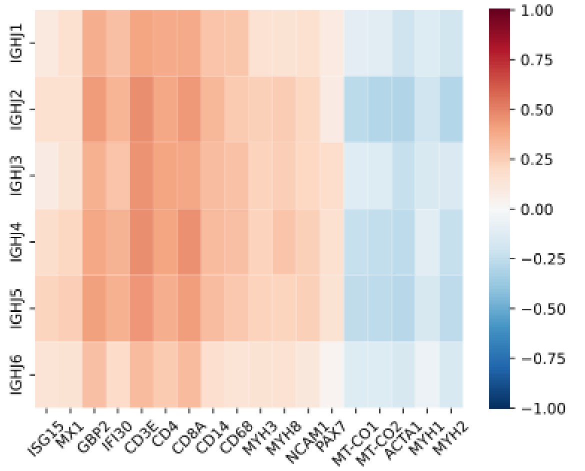

medRxiv preprint doi: https://doi.org/10.1101/2025.06.03.25328909; this version posted June 4, 2025. The copyright holder for this preprint (which was not certified by peer review) is the author/funder, who has granted medRxiv a license to display the preprint in perpetuity.
This article is a US Government work. It is not subject to copyright under 17 USC 105 and is also made available for use under a CC0 license.

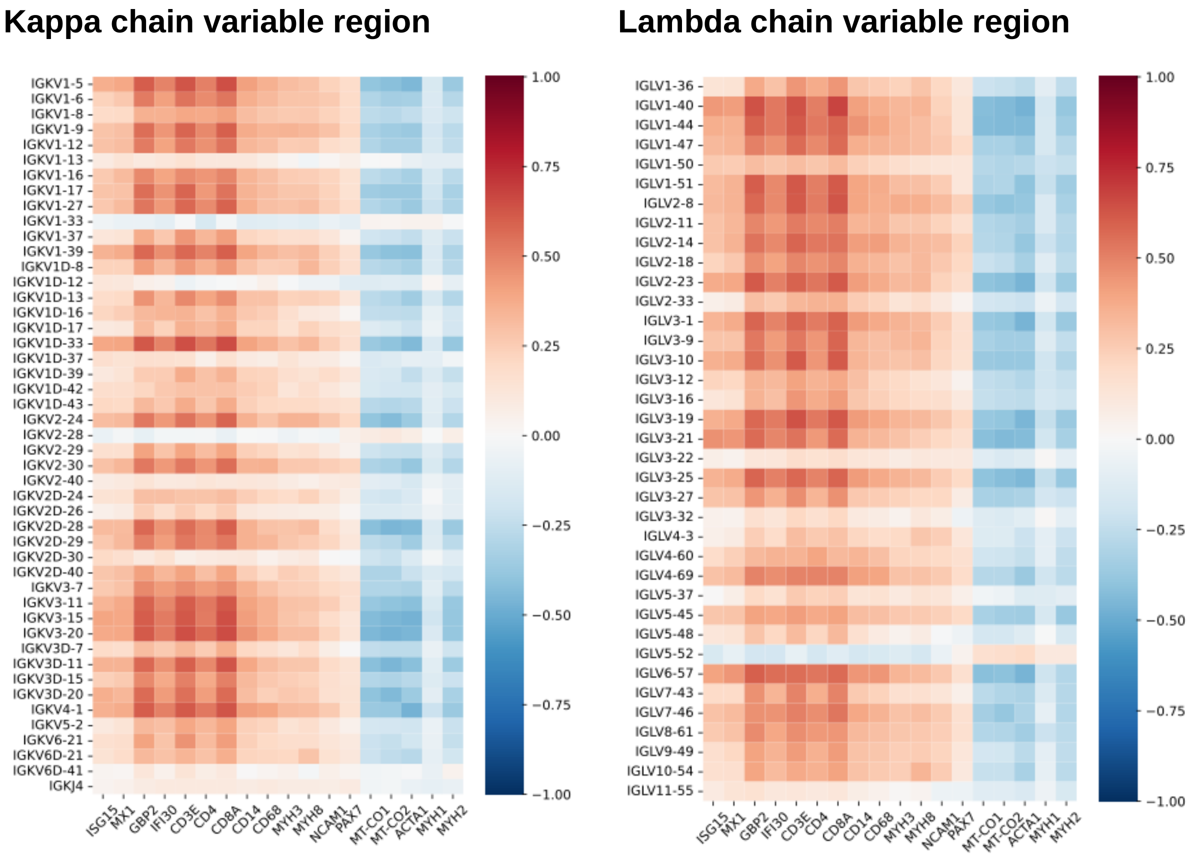

medRxiv preprint doi: https://doi.org/10.1101/2025.06.03.25328909; this version posted June 4, 2025. The copyright holder for this preprint (which was not certified by peer review) is the author/funder, who has granted medRxiv a license to display the preprint in perpetuity.
This article is a US Government work. It is not subject to copyright under 17 USC 105 and is also made available for use under a CC0 license.

## Supplementary Table 1. Distribution of groups included in the study.

| Group | N = 289[1] |
|---|---|
| NT | 37 (13%) |
| Anti-Mi2 | 22 (7.6%) |
| Anti-NXP2 | 21 (7.3%) |
| Anti-TIF1γ | 28 (9.7%) |
| Anti-MDA5 | 11 (3.8%) |
| Anti-Jo1 | 37 (13%) |
| Anti-HMGCR | 60 (21%) |
| Anti-SRP | 20 (6.9%) |
| IBM | 53 (18%) |

[1] n (%)

medRxiv preprint doi: https://doi.org/10.1101/2025.06.03.25328909; this version posted June 4, 2025. The copyright holder for this preprint (which was not certified by peer review) is the author/funder, who has granted medRxiv a license to display the preprint in perpetuity.
This article is a US Government work. It is not subject to copyright under 17 USC 105 and is also made available for use under a CC0 license.

**Supplementary Table 2. Expression of immunoglobulin kappa/lambda constant region genes across disease groups, shown as comparisons versus all other muscle biopsies (vs. ALL) and versus normal muscle biopsies (vs. NT).** DM: dermatomyositis; IMNM: immune-mediated necrotizing myopathy; IBM: inclusion body myositis; NT: normal muscle biopsy.

| DM vs. ALL | | | Mi2 vs. ALL | | | NXP2 vs. ALL | | | MDA5 vs. ALL | | | TIF1γvs. ALL | | |
|---|---|---|---|---|---|---|---|---|---|---|---|---|---|---|
| Gene | LogFC | q-val | Gene | LogFC | q-val | Gene | LogFC | q-val | Gene | LogFC | q-val | Gene | LogFC | q-val |
| IGLC6 | -0.7 | 0.024 | IGLC6 | -0.7 | 0.265 | IGLC7 | -1.3 | 0.208 | IGLC2 | 1.5 | 0.587 | IGLC6 | -0.6 | 0.372 |
| IGKC | -0.2 | 0.693 | IGKC | -0.4 | 0.691 | IGLC6 | -0.8 | 0.291 | IGLC7 | 1.2 | 0.701 | IGLC7 | 0.4 | 0.714 |
| IGLC7 | -0.2 | 0.767 | IGLC1 | 0.4 | 0.767 | IGKC | -1.0 | 0.481 | IGLC3 | 1.0 | 0.722 | IGKC | 0.3 | 0.762 |
| IGLC2 | 0.1 | 0.832 | IGLC2 | -0.3 | 0.775 | IGLC1 | -0.8 | 0.658 | IGKC | 0.8 | 0.739 | IGLC2 | 0.3 | 0.797 |
| IGLC3 | 0.1 | 0.840 | IGLC7 | -0.3 | 0.804 | IGLC3 | -0.5 | 0.740 | IGLC1 | 0.7 | 0.812 | IGLC3 | 0.3 | 0.835 |
| IGLC1 | -0.1 | 0.899 | IGLC3 | 0.0 | 0.995 | IGLC2 | -0.4 | 0.762 | IGLC6 | 0.2 | 0.939 | IGLC1 | -0.2 | 0.892 |

| JO1 vs. ALL | | | IMNM vs. ALL | | | HMGCR vs. ALL | | | SRP vs. ALL | | | IBM vs. ALL | | |
|---|---|---|---|---|---|---|---|---|---|---|---|---|---|---|
| Gene | LogFC | q-val | Gene | LogFC | q-val | Gene | LogFC | q-val | Gene | LogFC | q-val | Gene | LogFC | q-val |
| IGKC | 2.1 | 3.45e-04 | IGLC7 | -1.8 | 1.46e-04 | IGLC7 | -1.8 | 5.74e-04 | IGLC7 | -0.9 | 0.996 | IGKC | 3.8 | 1.26e-24 |
| IGLC3 | 2.2 | 8.79e-04 | IGKC | -1.9 | 0.001 | IGLC3 | -2.4 | 0.003 | IGKC | -0.7 | 0.996 | IGLC3 | 3.8 | 1.10e-20 |
| IGLC7 | 2.1 | 0.001 | IGLC3 | -2.2 | 0.001 | IGKC | -2.0 | 0.004 | IGLC6 | -0.3 | 0.996 | IGLC1 | 3.7 | 5.40e-16 |
| IGLC2 | 2.0 | 0.003 | IGLC2 | -1.8 | 0.003 | IGLC2 | -2.0 | 0.005 | IGLC3 | -0.4 | 0.996 | IGLC2 | 3.1 | 7.35e-15 |
| IGLC1 | 2.2 | 0.004 | IGLC6 | -0.8 | 0.012 | IGLC6 | -0.9 | 0.012 | IGLC2 | -0.3 | 0.996 | IGLC7 | 2.6 | 6.01e-09 |
| IGLC6 | 1.0 | 0.130 | IGLC1 | -1.8 | 0.017 | IGLC1 | -2.2 | 0.017 | IGLC1 | 0.0 | 0.999 | IGLC6 | 1.7 | 3.94e-07 |

| DM vs. NT | | | Mi2 vs. NT | | | NXP2 vs. NT | | | MDA5 vs. NT | | | TIF1γvs. NT | | |
|---|---|---|---|---|---|---|---|---|---|---|---|---|---|---|
| Gene | LogFC | q-val | Gene | LogFC | q-val | Gene | LogFC | q-val | Gene | LogFC | q-val | Gene | LogFC | q-val |
| IGKC | 3.1 | 6.28e-05 | IGLC3 | 3.6 | 7.39e-05 | IGLC3 | 3.1 | 1.79e-05 | IGLC2 | 4.5 | 2.31e-05 | IGKC | 3.5 | 9.68e-06 |
| IGLC3 | 3.7 | 7.30e-05 | IGKC | 2.8 | 2.46e-04 | IGLC2 | 2.7 | 9.99e-05 | IGKC | 3.9 | 1.64e-04 | IGLC2 | 3.3 | 2.84e-04 |
| IGLC2 | 3.2 | 2.25e-04 | IGLC1 | 3.9 | 2.49e-04 | IGKC | 2.3 | 1.75e-04 | IGLC3 | 4.5 | 8.89e-04 | IGLC7 | 2.2 | 5.43e-04 |
| IGLC7 | 1.7 | 3.31e-04 | IGLC2 | 2.8 | 0.003 | IGLC1 | 2.8 | 0.001 | IGLC7 | 2.9 | 0.001 | IGLC3 | 3.8 | 6.17e-04 |
| IGLC1 | 3.5 | 6.19e-04 | IGLC7 | 1.5 | 0.007 | IGLC7 | 0.6 | 0.154 | IGLC1 | 4.2 | 0.004 | IGLC1 | 3.4 | 0.003 |
| IGLC6 | 0.2 | 0.671 | IGLC6 | -0.1 | 0.906 | IGLC6 | -0.1 | 0.900 | IGLC6 | 0.7 | 0.321 | IGLC6 | 0.1 | 0.737 |

| JO1 vs. NT | | | IMNM vs. NT | | | HMGCR vs. NT | | | SRP vs. NT | | | IBM vs. NT | | |
|---|---|---|---|---|---|---|---|---|---|---|---|---|---|---|
| Gene | LogFC | q-val | Gene | LogFC | q-val | Gene | LogFC | q-val | Gene | LogFC | q-val | Gene | LogFC | q-val |
| IGLC7 | 3.7 | 2.53e-09 | IGKC | 1.8 | 0.002 | IGKC | 1.6 | 0.010 | IGLC1 | 3.5 | 2.12e-06 | IGLC7 | 4.0 | 1.92e-11 |
| IGKC | 5.1 | 2.49e-08 | IGLC1 | 2.2 | 0.009 | IGLC1 | 1.8 | 0.039 | IGLC2 | 2.8 | 6.68e-06 | IGLC1 | 6.6 | 1.17e-09 |
| IGLC3 | 5.5 | 3.81e-07 | IGLC2 | 1.8 | 0.010 | IGLC2 | 1.5 | 0.044 | IGLC3 | 3.2 | 1.07e-05 | IGKC | 6.4 | 1.45e-09 |
| IGLC2 | 4.8 | 5.07e-07 | IGLC3 | 2.0 | 0.011 | IGLC3 | 1.7 | 0.045 | IGKC | 2.6 | 2.23e-05 | IGLC6 | 2.1 | 1.77e-08 |
| IGLC1 | 5.5 | 2.68e-05 | IGLC7 | 0.6 | 0.109 | IGLC7 | 0.4 | 0.261 | IGLC7 | 1.0 | 0.051 | IGLC3 | 6.8 | 1.92e-08 |
| IGLC6 | 1.4 | 2.54e-04 | IGLC6 | 0.0 | 0.910 | IGLC6 | -0.1 | 0.813 | IGLC6 | 0.5 | 0.196 | IGLC2 | 5.8 | 5.41e-07 |

medRxiv preprint doi: https://doi.org/10.1101/2025.06.03.25328909; this version posted June 4, 2025. The copyright holder for this preprint (which was not certified by peer review) is the author/funder, who has granted medRxiv a license to display the preprint in perpetuity. This article is a US Government work. It is not subject to copyright under 17 USC 105 and is also made available for use under a CC0 license.

**Supplementary Table 3. Expression of top 10 immunoglobulin heavy chain variable region genes across disease groups, shown as comparisons versus all other muscle biopsies (vs. ALL) and versus normal muscle biopsies (vs. NT).** DM: dermatomyositis; IMNM: immune-mediated necrotizing myopathy; IBM: inclusion body myositis; NT: normal muscle biopsy.

| DM vs. ALL | | | Mi2 vs. ALL | | | NXP2 vs. ALL | | | MDA5 vs. ALL | | | TIF1γvs. ALL | | |
|---|---|---|---|---|---|---|---|---|---|---|---|---|---|---|
| Gene | LogFC | q-val | Gene | LogFC | q-val | Gene | LogFC | q-val | Gene | LogFC | q-val | Gene | LogFC | q-val |
| IGHV1-69-2 | -0.8 | 0.003 | IGHV3-38 | -1.1 | 0.009 | IGHV4-28 | -1.3 | 0.030 | IGHV5-51 | 1.8 | 0.448 | IGHV1-69-2 | -0.9 | 0.087 |
| IGHV3-38 | -0.6 | 0.006 | IGHV7-81 | -0.8 | 0.018 | IGHV1-24 | -1.4 | 0.034 | IGHV1-2 | 1.3 | 0.649 | IGHV1-45 | -0.5 | 0.177 |
| IGHV3-16 | -0.5 | 0.007 | IGHV4-61 | -1.2 | 0.032 | IGHV4-4 | -1.5 | 0.034 | IGHV1-24 | 1.1 | 0.694 | IGHV3-16 | -0.5 | 0.250 |
| IGHV1-45 | -0.4 | 0.012 | IGHV1-69-2 | -1.0 | 0.035 | IGHV3-20 | -1.5 | 0.035 | IGHV1-69 | 1.2 | 0.700 | IGHV1-24 | -0.8 | 0.259 |
| IGHV2-26 | -0.8 | 0.013 | IGHV3-35 | -0.8 | 0.039 | IGHV2-26 | -1.2 | 0.072 | IGHV2-5 | 1.1 | 0.701 | IGHV7-4-1 | -0.7 | 0.309 |
| IGHV7-81 | -0.4 | 0.018 | IGHV2-26 | -1.2 | 0.057 | IGHV1-69 | -1.5 | 0.084 | IGHV6-1 | 0.9 | 0.716 | IGHV1-69D | -0.9 | 0.313 |
| IGHV3-48 | -0.9 | 0.025 | IGHV1-45 | -0.6 | 0.079 | IGHV3-48 | -1.3 | 0.129 | IGHV3-33 | 1.1 | 0.717 | IGHV4-28 | -0.6 | 0.372 |
| IGHV3-35 | -0.5 | 0.027 | IGHV3-16 | -0.6 | 0.081 | IGHV1-58 | -0.8 | 0.130 | IGHV4-34 | 0.9 | 0.719 | IGHV3-13 | -0.5 | 0.383 |
| IGHV3-64D | -0.7 | 0.032 | IGHV3-64D | -0.9 | 0.111 | IGHV3-64D | -1.0 | 0.153 | IGHV3-13 | 0.8 | 0.719 | IGHV2-5 | -0.7 | 0.422 |
| IGHV3-43 | -0.8 | 0.032 | IGHV3-13 | -0.8 | 0.123 | IGHV4-59 | -1.7 | 0.168 | IGHV3-49 | 0.9 | 0.721 | IGHV4-61 | -0.5 | 0.516 |

| JO1 vs. ALL | | | IMNM vs. ALL | | | HMGCR vs. ALL | | | SRP vs. ALL | | | IBM vs. ALL | | |
|---|---|---|---|---|---|---|---|---|---|---|---|---|---|---|
| Gene | LogFC | q-val | Gene | LogFC | q-val | Gene | LogFC | q-val | Gene | LogFC | q-val | Gene | LogFC | q-val |
| IGHV3-23 | 2.8 | 9.65e-07 | IGHV3-73 | -1.2 | 3.31e-04 | IGHV3-53 | -1.5 | 5.68e-04 | IGHV3-35 | -0.6 | 0.928 | IGHV4-59 | 3.6 | 5.44e-19 |
| IGHV3-7 | 2.6 | 1.69e-06 | IGHV3-53 | -1.4 | 4.95e-04 | IGHV3-74 | -1.9 | 0.001 | IGHV3-64 | -0.8 | 0.972 | IGHV1-18 | 3.3 | 5.57e-16 |
| IGHV3-21 | 2.5 | 3.00e-05 | IGHV3-74 | -1.7 | 0.001 | IGHV3-7 | -2.0 | 0.001 | IGHV1-69-2 | -0.6 | 0.990 | IGHV3-30 | 3.5 | 1.12e-15 |
| IGHV3-48 | 2.3 | 6.20e-05 | IGHV3-21 | -1.7 | 0.001 | IGHV4-4 | -1.4 | 0.002 | IGHV6-1 | -0.8 | 0.994 | IGHV1-46 | 2.7 | 6.46e-13 |
| IGHV3-20 | 2.1 | 9.71e-05 | IGHV5-10-1 | -1.1 | 0.003 | IGHV3-66 | -1.3 | 0.002 | IGHV3-73 | -0.8 | 0.996 | IGHV3-23 | 3.1 | 1.01e-12 |
| IGHV3-74 | 2.2 | 1.74e-04 | IGHV3-7 | -1.6 | 0.003 | IGHV3-73 | -1.2 | 0.003 | IGHV4-61 | -0.7 | 0.996 | IGHV3-74 | 2.7 | 1.32e-11 |
| IGHV2-70 | 1.8 | 4.67e-04 | IGHV3-11 | -1.4 | 0.004 | IGHV4-59 | -2.0 | 0.003 | IGHV3-11 | -0.9 | 0.996 | IGHV3-15 | 2.7 | 1.96e-11 |
| IGHV3-53 | 2.1 | 4.84e-04 | IGHV3-66 | -1.1 | 0.004 | IGHV5-10-1 | -1.2 | 0.003 | IGHV7-81 | -0.3 | 0.996 | IGHV3-33 | 2.9 | 3.27e-11 |
| IGHV3-66 | 1.9 | 8.59e-04 | IGHV1-18 | -1.5 | 0.004 | IGHV5-51 | -1.7 | 0.003 | IGHV2-5 | -0.8 | 0.996 | IGHV4-39 | 2.6 | 4.82e-11 |
| IGHV3-49 | 2.0 | 0.001 | IGHV3-20 | -1.2 | 0.004 | IGHV3-49 | -1.4 | 0.004 | IGHV3-64D | -0.5 | 0.996 | IGHV3-7 | 2.7 | 1.41e-10 |

| DM vs. NT | | | Mi2 vs. NT | | | NXP2 vs. NT | | | MDA5 vs. NT | | | TIF1γvs. NT | | |
|---|---|---|---|---|---|---|---|---|---|---|---|---|---|---|
| Gene | LogFC | q-val | Gene | LogFC | q-val | Gene | LogFC | q-val | Gene | LogFC | q-val | Gene | LogFC | q-val |
| IGHV1-18 | 1.9 | 6.02e-05 | IGHV7-81 | -0.8 | 1.10e-05 | IGHV1-18 | 1.3 | 0.017 | IGHV5-51 | 3.4 | 5.79e-04 | IGHV3-53 | 1.9 | 2.18e-04 |
| IGHV3-7 | 2.1 | 1.94e-04 | IGHV3-38 | -0.8 | 3.64e-05 | IGHV3-64 | 1.1 | 0.036 | IGHV1-2 | 2.9 | 5.89e-04 | IGHV1-18 | 2.1 | 2.99e-04 |
| IGHV3-11 | 1.5 | 5.00e-04 | IGHV3-7 | 1.9 | 0.002 | IGHV3-7 | 1.4 | 0.049 | IGHV1-18 | 2.9 | 0.002 | IGHV3-7 | 2.5 | 4.92e-04 |
| IGHV3-53 | 1.3 | 0.001 | IGHV1-18 | 1.7 | 0.003 | IGHV4-39 | 1.1 | 0.072 | IGHV3-11 | 2.4 | 0.002 | IGHV4-59 | 2.1 | 0.002 |
| IGHV2-5 | 1.3 | 0.003 | IGHV4-34 | 1.5 | 0.004 | IGHV3-11 | 1.0 | 0.075 | IGHV3-7 | 2.9 | 0.003 | IGHV3-11 | 1.7 | 0.002 |
| IGHV4-34 | 1.3 | 0.004 | IGHV3-23 | 2.1 | 0.005 | IGHV7-81 | -0.5 | 0.102 | IGHV3-33 | 3.0 | 0.004 | IGHV3-23 | 2.6 | 0.002 |
| IGHV3-23 | 2.0 | 0.004 | IGHV1-69-2 | -0.9 | 0.006 | IGHV5-51 | 1.3 | 0.106 | IGHV4-59 | 2.8 | 0.004 | IGHV3-30 | 2.2 | 0.004 |
| IGHV4-59 | 1.8 | 0.004 | IGHV4-59 | 1.9 | 0.007 | IGHV2-5 | 1.0 | 0.111 | IGHV4-34 | 2.2 | 0.004 | IGHV1-69 | 1.4 | 0.007 |
| IGHV5-51 | 1.6 | 0.005 | IGHV3-74 | 1.4 | 0.007 | IGHV3-23 | 1.1 | 0.120 | IGHV2-5 | 2.6 | 0.006 | IGHV3-74 | 2.1 | 0.008 |
| IGHV3-30 | 1.9 | 0.007 | IGHV3-35 | -0.6 | 0.008 | IGHV4-34 | 0.9 | 0.127 | IGHV3-30 | 2.6 | 0.011 | IGHV1-69-2 | -0.8 | 0.011 |

| JO1 vs. NT | | | IMNM vs. NT | | | HMGCR vs. NT | | | SRP vs. NT | | | IBM vs. NT | | |
|---|---|---|---|---|---|---|---|---|---|---|---|---|---|---|
| Gene | LogFC | q-val | Gene | LogFC | q-val | Gene | LogFC | q-val | Gene | LogFC | q-val | Gene | LogFC | q-val |
| IGHV3-7 | 4.7 | 1.81e-10 | IGHV1-18 | 1.2 | 0.008 | IGHV1-18 | 1.0 | 0.037 | IGHV4-59 | 2.4 | 2.37e-04 | IGHV1-18 | 5.1 | 1.92e-13 |
| IGHV1-18 | 4.0 | 1.21e-09 | IGHV3-7 | 1.2 | 0.029 | IGHV3-23 | 1.0 | 0.127 | IGHV3-7 | 2.3 | 3.55e-04 | IGHV1-46 | 3.8 | 4.21e-13 |
| IGHV3-20 | 3.0 | 1.22e-09 | IGHV3-23 | 1.3 | 0.044 | IGHV3-11 | 0.7 | 0.140 | IGHV3-23 | 2.3 | 0.002 | IGHV3-7 | 4.7 | 1.66e-12 |
| IGHV3-48 | 3.4 | 1.77e-09 | IGHV1-2 | 0.9 | 0.062 | IGHV3-30 | 1.0 | 0.142 | IGHV1-18 | 1.7 | 0.007 | IGHV3-43 | 3.0 | 1.94e-12 |
| IGHV3-21 | 3.9 | 6.18e-08 | IGHV3-48 | 0.8 | 0.084 | IGHV3-7 | 0.8 | 0.156 | IGHV1-2 | 1.7 | 0.008 | IGHV3-53 | 3.2 | 2.83e-12 |
| IGHV2-5 | 3.1 | 6.49e-08 | IGHV3-30 | 1.1 | 0.096 | IGHV2-5 | 0.6 | 0.199 | IGHV5-51 | 1.7 | 0.009 | IGHV4-34 | 3.5 | 4.27e-12 |
| IGHV1-46 | 2.8 | 1.87e-07 | IGHV3-11 | 0.7 | 0.099 | IGHV3-38 | -0.3 | 0.216 | IGHV2-70D | 1.2 | 0.009 | IGHV2-5 | 3.5 | 2.41e-11 |
| IGHV3-11 | 3.2 | 1.91e-07 | IGHV1-46 | 0.7 | 0.103 | IGHV7-81 | -0.3 | 0.221 | IGHV3-74 | 1.5 | 0.017 | IGHV3-30 | 5.3 | 4.45e-11 |
| IGHV3-43 | 2.6 | 2.02e-07 | IGHV4-59 | 1.0 | 0.105 | IGHV1-2 | 0.6 | 0.223 | IGHV3-43 | 1.1 | 0.030 | IGHV3-11 | 4.0 | 8.47e-11 |
| IGHV3-49 | 3.0 | 2.90e-07 | IGHV7-81 | -0.3 | 0.114 | IGHV3-48 | 0.6 | 0.237 | IGHV2-70 | 0.8 | 0.031 | IGHV3-48 | 3.4 | 9.50e-11 |

medRxiv preprint doi: https://doi.org/10.1101/2025.06.03.25328909; this version posted June 4, 2025. The copyright holder for this preprint (which was not certified by peer review) is the author/funder, who has granted medRxiv a license to display the preprint in perpetuity. This article is a US Government work. It is not subject to copyright under 17 USC 105 and is also made available for use under a CC0 license.

**Supplementary Table 4. Expression of immunoglobulin heavy chain joining region genes across disease groups, shown as comparisons versus all other muscle biopsies (vs. ALL) and versus normal muscle biopsies (vs. NT).** DM: dermatomyositis; IMNM: immune-mediated necrotizing myopathy; IBM: inclusion body myositis; NT: normal muscle biopsy.

| DM vs. ALL | | | Mi2 vs. ALL | | | NXP2 vs. ALL | | | MDA5 vs. ALL | | | TIF1γvs. ALL | | |
|---|---|---|---|---|---|---|---|---|---|---|---|---|---|---|
| Gene | LogFC | q-val | Gene | LogFC | q-val | Gene | LogFC | q-val | Gene | LogFC | q-val | Gene | LogFC | q-val |
| IGHJ3 | -0.8 | 0.001 | IGHJ2 | -0.8 | 0.034 | IGHJ2 | -0.7 | 0.165 | IGHJ6 | -0.4 | 0.767 | IGHJ3 | -0.6 | 0.204 |
| IGHJ2 | -0.6 | 0.010 | IGHJ3 | -0.8 | 0.067 | IGHJ3 | -0.7 | 0.191 | IGHJ2 | 0.4 | 0.795 | IGHJ1 | -0.4 | 0.298 |
| IGHJ1 | -0.4 | 0.014 | IGHJ1 | -0.6 | 0.117 | IGHJ5 | -0.6 | 0.248 | IGHJ5 | 0.3 | 0.856 | IGHJ2 | -0.4 | 0.455 |
| IGHJ6 | -0.6 | 0.024 | IGHJ4 | -0.6 | 0.204 | IGHJ6 | -0.5 | 0.439 | IGHJ1 | 0.1 | 0.959 | IGHJ5 | -0.4 | 0.476 |
| IGHJ5 | -0.4 | 0.075 | IGHJ6 | -0.6 | 0.244 | IGHJ1 | -0.4 | 0.449 | IGHJ3 | -0.1 | 0.961 | IGHJ6 | -0.4 | 0.578 |
| IGHJ4 | -0.4 | 0.087 | IGHJ5 | -0.4 | 0.395 | IGHJ4 | -0.4 | 0.586 | IGHJ4 | 0.1 | 0.976 | IGHJ4 | -0.3 | 0.626 |

| JO1 vs. ALL | | | IMNM vs. ALL | | | HMGCR vs. ALL | | | SRP vs. ALL | | | IBM vs. ALL | | |
|---|---|---|---|---|---|---|---|---|---|---|---|---|---|---|
| Gene | LogFC | q-val | Gene | LogFC | q-val | Gene | LogFC | q-val | Gene | LogFC | q-val | Gene | LogFC | q-val |
| IGHJ5 | 1.0 | 0.053 | IGHJ4 | -0.5 | 0.040 | IGHJ4 | -0.6 | 0.050 | IGHJ6 | -0.6 | 0.991 | IGHJ2 | 1.0 | 1.16e-04 |
| IGHJ3 | 0.9 | 0.057 | IGHJ2 | -0.5 | 0.064 | IGHJ5 | -0.5 | 0.171 | IGHJ2 | -0.4 | 0.996 | IGHJ6 | 1.2 | 3.96e-04 |
| IGHJ2 | 0.7 | 0.202 | IGHJ6 | -0.5 | 0.108 | IGHJ2 | -0.4 | 0.178 | IGHJ1 | -0.3 | 0.996 | IGHJ4 | 1.0 | 0.001 |
| IGHJ4 | 0.7 | 0.216 | IGHJ1 | -0.3 | 0.123 | IGHJ1 | -0.3 | 0.248 | IGHJ4 | -0.2 | 0.996 | IGHJ1 | 0.5 | 0.020 |
| IGHJ1 | 0.5 | 0.238 | IGHJ5 | -0.4 | 0.158 | IGHJ6 | -0.4 | 0.368 | IGHJ5 | -0.1 | 0.996 | IGHJ3 | 0.7 | 0.036 |
| IGHJ6 | 0.5 | 0.529 | IGHJ3 | -0.2 | 0.563 | IGHJ3 | -0.3 | 0.444 | IGHJ3 | 0.1 | 0.996 | IGHJ5 | 0.6 | 0.064 |

| DM vs. NT | | | Mi2 vs. NT | | | NXP2 vs. NT | | | MDA5 vs. NT | | | TIF1γvs. NT | | |
|---|---|---|---|---|---|---|---|---|---|---|---|---|---|---|
| Gene | LogFC | q-val | Gene | LogFC | q-val | Gene | LogFC | q-val | Gene | LogFC | q-val | Gene | LogFC | q-val |
| IGHJ1 | -0.3 | 0.145 | IGHJ1 | -0.5 | 0.029 | IGHJ3 | -0.5 | 0.213 | IGHJ2 | 0.6 | 0.167 | IGHJ1 | -0.4 | 0.175 |
| IGHJ3 | -0.4 | 0.196 | IGHJ2 | -0.5 | 0.034 | IGHJ1 | -0.4 | 0.233 | IGHJ5 | 0.5 | 0.355 | IGHJ3 | -0.4 | 0.291 |
| IGHJ2 | -0.1 | 0.696 | IGHJ3 | -0.6 | 0.048 | IGHJ2 | -0.4 | 0.278 | IGHJ4 | 0.4 | 0.516 | IGHJ4 | 0.2 | 0.638 |
| IGHJ6 | -0.1 | 0.697 | IGHJ4 | -0.2 | 0.443 | IGHJ5 | -0.4 | 0.347 | IGHJ6 | -0.2 | 0.714 | IGHJ5 | -0.1 | 0.889 |
| IGHJ4 | 0.0 | 0.887 | IGHJ6 | -0.2 | 0.528 | IGHJ6 | -0.2 | 0.645 | IGHJ3 | 0.1 | 0.899 | IGHJ2 | 0.0 | 0.942 |
| IGHJ5 | 0.0 | 0.901 | IGHJ5 | -0.2 | 0.609 | IGHJ4 | 0.0 | 0.956 | IGHJ1 | 0.0 | 0.953 | IGHJ6 | 0.0 | 0.974 |

| JO1 vs. NT | | | IMNM vs. NT | | | HMGCR vs. NT | | | SRP vs. NT | | | IBM vs. NT | | |
|---|---|---|---|---|---|---|---|---|---|---|---|---|---|---|
| Gene | LogFC | q-val | Gene | LogFC | q-val | Gene | LogFC | q-val | Gene | LogFC | q-val | Gene | LogFC | q-val |
| IGHJ5 | 1.0 | 0.001 | IGHJ1 | -0.3 | 0.163 | IGHJ1 | -0.3 | 0.224 | IGHJ3 | 0.5 | 0.207 | IGHJ2 | 1.2 | 5.96e-06 |
| IGHJ2 | 0.8 | 0.002 | IGHJ6 | -0.1 | 0.802 | IGHJ4 | -0.2 | 0.620 | IGHJ1 | -0.2 | 0.345 | IGHJ4 | 1.2 | 4.87e-05 |
| IGHJ4 | 0.9 | 0.003 | IGHJ4 | -0.1 | 0.802 | IGHJ5 | -0.1 | 0.743 | IGHJ6 | -0.2 | 0.538 | IGHJ6 | 1.3 | 4.24e-04 |
| IGHJ3 | 1.0 | 0.017 | IGHJ2 | -0.1 | 0.822 | IGHJ2 | -0.1 | 0.846 | IGHJ4 | 0.2 | 0.599 | IGHJ3 | 0.8 | 0.005 |
| IGHJ6 | 0.7 | 0.052 | IGHJ3 | 0.0 | 0.906 | IGHJ3 | -0.1 | 0.869 | IGHJ5 | 0.2 | 0.618 | IGHJ5 | 0.8 | 0.008 |
| IGHJ1 | 0.4 | 0.127 | IGHJ5 | 0.0 | 0.907 | IGHJ6 | 0.0 | 0.973 | IGHJ2 | 0.1 | 0.848 | IGHJ1 | 0.5 | 0.049 |

medRxiv preprint doi: https://doi.org/10.1101/2025.06.03.25328909; this version posted June 4, 2025. The copyright holder for this preprint (which was not certified by peer review) is the author/funder, who has granted medRxiv a license to display the preprint in perpetuity. This article is a US Government work. It is not subject to copyright under 17 USC 105 and is also made available for use under a CC0 license.

**Supplementary Table 5. Expression of top 10 immunoglobulin kappa variable region genes across disease groups, shown as comparisons versus all other muscle biopsies (vs. ALL) and versus normal muscle biopsies (vs. NT).** DM: dermatomyositis; IMNM: immune-mediated necrotizing myopathy; IBM: inclusion body myositis; NT: normal muscle biopsy.

| DM vs. ALL | | | Mi2 vs. ALL | | | NXP2 vs. ALL | | | MDA5 vs. ALL | | | TIF1γvs. ALL | | |
|---|---|---|---|---|---|---|---|---|---|---|---|---|---|---|
| Gene | LogFC | q-val | Gene | LogFC | q-val | Gene | LogFC | q-val | Gene | LogFC | q-val | Gene | LogFC | q-val |
| IGKV1D-17 | -0.8 | 8.15e-04 | IGKV2-28 | -0.9 | 0.016 | IGKV6D-21 | -1.0 | 0.072 | IGKV3D-20 | 1.1 | 0.708 | IGKV1D-17 | -0.9 | 0.058 |
| IGKV6-21 | -0.9 | 0.002 | IGKV2D-24 | -1.1 | 0.016 | IGKV2D-40 | -1.1 | 0.077 | IGKV1-39 | 1.2 | 0.715 | IGKV1D-16 | -0.8 | 0.201 |
| IGKV2-29 | -0.7 | 0.004 | IGKV1D-12 | -0.8 | 0.018 | IGKV3-7 | -1.2 | 0.079 | IGKV1D-12 | -0.4 | 0.717 | IGKV1D-43 | -0.6 | 0.241 |
| IGKV1-37 | -0.6 | 0.013 | IGKV6-21 | -1.2 | 0.023 | IGKV1-27 | -1.5 | 0.080 | IGKV1D-16 | 0.9 | 0.718 | IGKV5-2 | -0.6 | 0.259 |
| IGKV5-2 | -0.6 | 0.019 | IGKV2-29 | -1.0 | 0.025 | IGKV1D-8 | -0.9 | 0.135 | IGKV2-30 | 1.0 | 0.719 | IGKV2-28 | -0.4 | 0.280 |
| IGKV1D-12 | -0.4 | 0.023 | IGKV1-37 | -1.0 | 0.027 | IGKV2-24 | -1.3 | 0.188 | IGKV3-11 | 1.0 | 0.721 | IGKV2-29 | -0.5 | 0.304 |
| IGKV1D-13 | -0.8 | 0.025 | IGKV1D-17 | -0.8 | 0.072 | IGKV1-9 | -1.5 | 0.190 | IGKV1-17 | 1.0 | 0.721 | IGKV6-21 | -0.6 | 0.310 |
| IGKV3D-15 | -0.6 | 0.032 | IGKV3D-15 | -0.8 | 0.150 | IGKV1-37 | -0.7 | 0.199 | IGKV1-13 | -0.5 | 0.722 | IGKV3D-15 | -0.5 | 0.420 |
| IGKV2D-29 | -0.8 | 0.033 | IGKV2D-29 | -1.0 | 0.155 | IGKV2D-28 | -1.7 | 0.210 | IGKV2-40 | -0.3 | 0.723 | IGKV1D-39 | -0.6 | 0.440 |
| IGKV1-17 | -1.0 | 0.038 | IGKV1D-13 | -1.0 | 0.167 | IGKV1-17 | -1.3 | 0.218 | IGKV1D-43 | 0.6 | 0.723 | IGKV1D-12 | -0.3 | 0.503 |

| JO1 vs. ALL | | | IMNM vs. ALL | | | HMGCR vs. ALL | | | SRP vs. ALL | | | IBM vs. ALL | | |
|---|---|---|---|---|---|---|---|---|---|---|---|---|---|---|
| Gene | LogFC | q-val | Gene | LogFC | q-val | Gene | LogFC | q-val | Gene | LogFC | q-val | Gene | LogFC | q-val |
| IGKV4-1 | 2.5 | 2.92e-05 | IGKV1D-33 | -2.0 | 6.18e-04 | IGKV1-12 | -2.1 | 1.71e-04 | IGKV3D-11 | -1.3 | 0.996 | IGKV2D-28 | 4.3 | 1.38e-28 |
| IGKV3-20 | 2.4 | 1.33e-04 | IGKV3D-11 | -1.9 | 6.32e-04 | IGKV1D-33 | -2.2 | 0.001 | IGKV3-7 | -0.7 | 0.996 | IGKV3-11 | 3.6 | 1.10e-19 |
| IGKV3D-11 | 2.2 | 7.40e-04 | IGKV1-12 | -1.8 | 6.36e-04 | IGKV2D-28 | -2.3 | 0.002 | IGKV1D-17 | 0.6 | 0.996 | IGKV3-20 | 3.5 | 4.24e-18 |
| IGKV1-27 | 2.0 | 0.002 | IGKV3-15 | -2.1 | 6.71e-04 | IGKV2-30 | -1.6 | 0.002 | IGKV1D-13 | -0.7 | 0.996 | IGKV1D-33 | 3.5 | 4.84e-18 |
| IGKV3-15 | 2.1 | 0.004 | IGKV2D-28 | -2.1 | 7.87e-04 | IGKV3-15 | -2.2 | 0.002 | IGKV1-27 | -0.8 | 0.996 | IGKV3-15 | 3.5 | 8.57e-17 |
| IGKV1-5 | 1.9 | 0.004 | IGKV3-11 | -1.9 | 8.77e-04 | IGKV1-5 | -2.0 | 0.002 | IGKV3-11 | -1.1 | 0.996 | IGKV1-9 | 3.4 | 1.27e-16 |
| IGKV3-11 | 1.9 | 0.006 | IGKV1-5 | -1.8 | 0.001 | IGKV3D-20 | -1.5 | 0.003 | IGKV2D-29 | -0.7 | 0.996 | IGKV1-39 | 3.6 | 2.54e-16 |
| IGKV3D-20 | 1.8 | 0.008 | IGKV1-27 | -1.4 | 0.002 | IGKV1-9 | -1.8 | 0.003 | IGKV1-8 | -0.5 | 0.996 | IGKV3D-11 | 3.4 | 1.06e-15 |
| IGKV6D-21 | 1.4 | 0.010 | IGKV3D-20 | -1.4 | 0.002 | IGKV3-20 | -2.2 | 0.004 | IGKV1-17 | -0.9 | 0.996 | IGKV1-5 | 3.1 | 2.03e-14 |
| IGKV1D-33 | 1.8 | 0.017 | IGKV3-20 | -1.9 | 0.002 | IGKV1-39 | -2.1 | 0.005 | IGKV2D-24 | -0.4 | 0.996 | IGKV1-6 | 2.9 | 2.29e-14 |

| DM vs. NT | | | Mi2 vs. NT | | | NXP2 vs. NT | | | MDA5 vs. NT | | | TIF1γvs. NT | | |
|---|---|---|---|---|---|---|---|---|---|---|---|---|---|---|
| Gene | LogFC | q-val | Gene | LogFC | q-val | Gene | LogFC | q-val | Gene | LogFC | q-val | Gene | LogFC | q-val |
| IGKV4-1 | 2.7 | 8.76e-05 | IGKV1D-12 | -0.9 | 5.75e-07 | IGKV4-1 | 2.1 | 6.18e-04 | IGKV1-39 | 3.8 | 4.57e-04 | IGKV2D-28 | 3.3 | 6.89e-06 |
| IGKV1-39 | 2.6 | 9.37e-05 | IGKV2-28 | -1.3 | 2.17e-06 | IGKV1-5 | 1.9 | 0.003 | IGKV3D-20 | 2.7 | 6.54e-04 | IGKV1-39 | 2.9 | 3.40e-05 |
| IGKV2D-28 | 2.5 | 1.75e-04 | IGKV1D-33 | 2.6 | 9.26e-06 | IGKV1D-33 | 1.8 | 0.009 | IGKV4-1 | 3.2 | 0.002 | IGKV3-15 | 2.9 | 1.11e-04 |
| IGKV3-15 | 2.5 | 1.95e-04 | IGKV3-15 | 2.6 | 1.43e-04 | IGKV2-30 | 1.2 | 0.028 | IGKV3-11 | 3.2 | 0.002 | IGKV4-1 | 3.0 | 3.28e-04 |
| IGKV3-20 | 2.6 | 2.54e-04 | IGKV3-20 | 2.8 | 2.13e-04 | IGKV3-15 | 1.6 | 0.034 | IGKV1D-12 | -0.6 | 0.002 | IGKV3-20 | 3.0 | 3.49e-04 |
| IGKV1D-33 | 2.2 | 2.85e-04 | IGKV2D-28 | 2.6 | 5.69e-04 | IGKV3-20 | 1.6 | 0.040 | IGKV1D-33 | 3.1 | 0.002 | IGKV1-5 | 2.4 | 6.73e-04 |
| IGKV3D-11 | 1.9 | 3.93e-04 | IGKV4-1 | 2.7 | 7.97e-04 | IGKV1-39 | 1.5 | 0.059 | IGKV3D-11 | 2.9 | 0.002 | IGKV3D-20 | 1.7 | 0.001 |
| IGKV3D-20 | 1.4 | 4.91e-04 | IGKV1-39 | 2.6 | 0.001 | IGKV3D-11 | 1.1 | 0.079 | IGKV2-30 | 2.5 | 0.003 | IGKV3D-11 | 2.1 | 0.001 |
| IGKV1-5 | 2.2 | 5.69e-04 | IGKV2D-24 | -0.7 | 0.002 | IGKV3-11 | 1.2 | 0.087 | IGKV1-17 | 2.6 | 0.003 | IGKV1-27 | 1.6 | 0.004 |
| IGKV3-11 | 2.0 | 9.52e-04 | IGKV3-11 | 2.2 | 0.002 | IGKV6D-21 | -0.7 | 0.087 | IGKV3-15 | 2.9 | 0.006 | IGKV2-30 | 1.6 | 0.006 |

| JO1 vs. NT | | | IMNM vs. NT | | | HMGCR vs. NT | | | SRP vs. NT | | | IBM vs. NT | | |
|---|---|---|---|---|---|---|---|---|---|---|---|---|---|---|
| Gene | LogFC | q-val | Gene | LogFC | q-val | Gene | LogFC | q-val | Gene | LogFC | q-val | Gene | LogFC | q-val |
| IGKV2-24 | 3.1 | 8.26e-09 | IGKV4-1 | 1.7 | 0.011 | IGKV4-1 | 1.5 | 0.030 | IGKV4-1 | 2.3 | 9.98e-04 | IGKV2D-28 | 6.3 | 2.86e-15 |
| IGKV3D-20 | 3.2 | 9.68e-09 | IGKV1-39 | 1.4 | 0.028 | IGKV1-17 | 0.9 | 0.047 | IGKV3-20 | 2.4 | 0.001 | IGKV1-27 | 3.9 | 3.74e-14 |
| IGKV2-30 | 3.1 | 1.86e-08 | IGKV3-20 | 1.5 | 0.046 | IGKV1-39 | 1.1 | 0.102 | IGKV1-39 | 2.4 | 0.002 | IGKV3-11 | 5.3 | 1.05e-12 |
| IGKV3-11 | 4.0 | 2.69e-08 | IGKV1-17 | 0.9 | 0.047 | IGKV3-20 | 1.2 | 0.126 | IGKV2D-28 | 2.2 | 0.009 | IGKV3D-20 | 3.9 | 2.67e-12 |
| IGKV3D-11 | 4.1 | 5.59e-08 | IGKV2D-28 | 1.3 | 0.050 | IGKV2D-28 | 1.0 | 0.141 | IGKV1-5 | 1.8 | 0.010 | IGKV1-9 | 4.8 | 1.35e-11 |
| IGKV1-27 | 3.3 | 2.39e-07 | IGKV2-24 | 0.8 | 0.065 | IGKV5-2 | -0.4 | 0.174 | IGKV1D-33 | 1.8 | 0.012 | IGKV2-24 | 4.0 | 3.95e-11 |
| IGKV1-9 | 3.3 | 2.55e-07 | IGKV3D-20 | 0.7 | 0.069 | IGKV3-11 | 0.8 | 0.174 | IGKV3-15 | 1.9 | 0.012 | IGKV2-30 | 3.8 | 4.53e-11 |
| IGKV3-15 | 4.4 | 4.58e-07 | IGKV3-15 | 1.1 | 0.087 | IGKV3D-11 | 0.8 | 0.184 | IGKV2-30 | 1.5 | 0.020 | IGKV1-17 | 4.4 | 8.92e-11 |
| IGKV4-1 | 5.0 | 6.64e-07 | IGKV1D-33 | 0.9 | 0.091 | IGKV2D-30 | -0.4 | 0.186 | IGKV2-24 | 1.4 | 0.021 | IGKV1D-33 | 5.3 | 1.10e-10 |
| IGKV3-20 | 4.9 | 8.59e-07 | IGKV3-11 | 0.9 | 0.100 | IGKV3-15 | 0.9 | 0.203 | IGKV3D-20 | 1.1 | 0.023 | IGKV1-16 | 3.4 | 2.56e-10 |

medRxiv preprint doi: https://doi.org/10.1101/2025.06.03.25328909; this version posted June 4, 2025. The copyright holder for this preprint (which was not certified by peer review) is the author/funder, who has granted medRxiv a license to display the preprint in perpetuity. This article is a US Government work. It is not subject to copyright under 17 USC 105 and is also made available for use under a CC0 license.

**Supplementary Table 6. Expression of top 10 immunoglobulin lambda variable region genes across disease groups, shown as comparisons versus all other muscle biopsies (vs. ALL) and versus normal muscle biopsies (vs. NT).** DM: dermatomyositis; IMNM: immune-mediated necrotizing myopathy; IBM: inclusion body myositis; NT: normal muscle biopsy.

| DM vs. ALL | | | Mi2 vs. ALL | | | NXP2 vs. ALL | | | MDA5 vs. ALL | | | TIF1γvs. ALL | | |
|---|---|---|---|---|---|---|---|---|---|---|---|---|---|---|
| Gene | LogFC | q-val | Gene | LogFC | q-val | Gene | LogFC | q-val | Gene | LogFC | q-val | Gene | LogFC | q-val |
| IGLV4-3 | -0.6 | 2.13e-04 | IGLV5-37 | -0.9 | 0.012 | IGLV3-9 | -1.5 | 0.068 | IGLV3-21 | 2.0 | 0.406 | IGLV5-37 | -0.8 | 0.056 |
| IGLV5-37 | -0.6 | 9.71e-04 | IGLV3-32 | -0.7 | 0.017 | IGLV5-52 | 1.2 | 0.116 | IGLV7-43 | 1.3 | 0.614 | IGLV4-3 | -0.6 | 0.075 |
| IGLV3-32 | -0.5 | 9.71e-04 | IGLV4-3 | -0.7 | 0.025 | IGLV3-1 | -1.3 | 0.254 | IGLV2-11 | 1.5 | 0.632 | IGLV10-54 | -0.9 | 0.107 |
| IGLV5-48 | -0.6 | 0.001 | IGLV2-33 | -0.8 | 0.040 | IGLV4-69 | -1.0 | 0.279 | IGLV1-51 | 1.6 | 0.667 | IGLV3-22 | -0.5 | 0.127 |
| IGLV2-33 | -0.6 | 0.003 | IGLV3-22 | -0.6 | 0.048 | IGLV3-25 | -1.2 | 0.295 | IGLV3-16 | 0.9 | 0.670 | IGLV5-48 | -0.6 | 0.131 |
| IGLV3-22 | -0.4 | 0.005 | IGLV7-43 | -1.1 | 0.073 | IGLV2-8 | -1.3 | 0.336 | IGLV6-57 | 1.1 | 0.692 | IGLV5-52 | -1.0 | 0.147 |
| IGLV10-54 | -0.7 | 0.015 | IGLV5-48 | -0.6 | 0.073 | IGLV1-44 | -1.2 | 0.356 | IGLV1-47 | 1.1 | 0.701 | IGLV3-32 | -0.4 | 0.156 |
| IGLV3-16 | -0.4 | 0.050 | IGLV3-16 | -0.7 | 0.089 | IGLV10-54 | -0.6 | 0.377 | IGLV3-27 | 0.8 | 0.705 | IGLV8-61 | -1.0 | 0.182 |
| IGLV7-43 | -0.6 | 0.072 | IGLV3-12 | -0.7 | 0.110 | IGLV5-48 | -0.4 | 0.384 | IGLV3-10 | 1.1 | 0.706 | IGLV3-16 | -0.6 | 0.194 |
| IGLV4-60 | -0.5 | 0.081 | IGLV4-60 | -0.7 | 0.224 | IGLV4-60 | -0.6 | 0.390 | IGLV1-44 | 1.1 | 0.708 | IGLV7-43 | -0.8 | 0.220 |

| JO1 vs. ALL | | | IMNM vs. ALL | | | HMGCR vs. ALL | | | SRP vs. ALL | | | IBM vs. ALL | | |
|---|---|---|---|---|---|---|---|---|---|---|---|---|---|---|
| Gene | LogFC | q-val | Gene | LogFC | q-val | Gene | LogFC | q-val | Gene | LogFC | q-val | Gene | LogFC | q-val |
| IGLV3-25 | 2.3 | 6.68e-05 | IGLV3-21 | -2.2 | 6.82e-05 | IGLV3-21 | -2.3 | 1.82e-04 | IGLV3-10 | -1.2 | 0.811 | IGLV2-23 | 3.7 | 1.68e-19 |
| IGLV1-44 | 2.0 | 0.001 | IGLV1-40 | -2.2 | 9.20e-05 | IGLV3-19 | -2.1 | 2.80e-04 | IGLV1-51 | -1.3 | 0.993 | IGLV2-8 | 3.4 | 2.74e-17 |
| IGLV1-40 | 2.1 | 0.002 | IGLV3-10 | -1.5 | 9.75e-05 | IGLV3-25 | -1.9 | 3.33e-04 | IGLV3-16 | -0.5 | 0.996 | IGLV1-40 | 3.4 | 7.54e-16 |
| IGLV3-19 | 2.0 | 0.002 | IGLV3-19 | -1.9 | 1.33e-04 | IGLV1-40 | -2.4 | 3.89e-04 | IGLV1-50 | -0.4 | 0.996 | IGLV1-51 | 3.6 | 4.03e-15 |
| IGLV3-1 | 2.0 | 0.002 | IGLV3-25 | -1.6 | 7.48e-04 | IGLV2-11 | -2.1 | 8.41e-04 | IGLV3-27 | -0.5 | 0.996 | IGLV1-44 | 2.9 | 1.30e-12 |
| IGLV6-57 | 1.8 | 0.003 | IGLV6-57 | -1.5 | 0.001 | IGLV2-23 | -2.3 | 0.001 | IGLV5-37 | -0.3 | 0.996 | IGLV2-14 | 2.9 | 1.54e-11 |
| IGLV2-8 | 2.0 | 0.003 | IGLV1-51 | -1.8 | 0.001 | IGLV6-57 | -1.7 | 0.001 | IGLV1-40 | -1.0 | 0.996 | IGLV3-1 | 2.8 | 1.84e-11 |
| IGLV2-11 | 1.9 | 0.006 | IGLV2-23 | -1.9 | 0.002 | IGLV1-44 | -2.1 | 0.001 | IGLV9-49 | -0.4 | 0.996 | IGLV3-19 | 2.8 | 3.55e-11 |
| IGLV2-23 | 1.9 | 0.007 | IGLV2-11 | -1.7 | 0.002 | IGLV2-8 | -2.0 | 0.003 | IGLV8-61 | -0.6 | 0.996 | IGLV3-9 | 2.6 | 3.42e-10 |
| IGLV4-60 | 1.4 | 0.009 | IGLV2-8 | -1.8 | 0.002 | IGLV3-9 | -1.4 | 0.003 | IGLV3-19 | -0.8 | 0.996 | IGLV6-57 | 2.4 | 4.10e-10 |

| DM vs. NT | | | Mi2 vs. NT | | | NXP2 vs. NT | | | MDA5 vs. NT | | | TIF1γvs. NT | | |
|---|---|---|---|---|---|---|---|---|---|---|---|---|---|---|
| Gene | LogFC | q-val | Gene | LogFC | q-val | Gene | LogFC | q-val | Gene | LogFC | q-val | Gene | LogFC | q-val |
| IGLV6-57 | 2.4 | 1.45e-07 | IGLV1-40 | 3.4 | 3.46e-07 | IGLV6-57 | 1.8 | 1.11e-04 | IGLV6-57 | 3.3 | 1.01e-04 | IGLV6-57 | 2.7 | 1.60e-06 |
| IGLV1-40 | 2.9 | 6.71e-07 | IGLV3-32 | -0.9 | 5.75e-07 | IGLV2-23 | 2.4 | 4.58e-04 | IGLV3-21 | 4.0 | 5.12e-04 | IGLV1-40 | 3.0 | 3.34e-06 |
| IGLV2-23 | 2.8 | 1.96e-05 | IGLV5-37 | -1.1 | 4.85e-06 | IGLV3-21 | 2.0 | 0.003 | IGLV2-11 | 3.5 | 5.67e-04 | IGLV1-44 | 2.8 | 3.35e-05 |
| IGLV1-44 | 2.6 | 2.15e-05 | IGLV4-3 | -0.8 | 8.83e-06 | IGLV1-40 | 2.0 | 0.004 | IGLV1-51 | 3.4 | 9.35e-04 | IGLV3-25 | 2.2 | 6.12e-05 |
| IGLV3-21 | 2.5 | 9.46e-05 | IGLV3-22 | -0.8 | 1.44e-05 | IGLV2-14 | 2.2 | 0.009 | IGLV1-40 | 3.6 | 0.001 | IGLV2-23 | 2.9 | 1.71e-04 |
| IGLV3-25 | 1.8 | 2.09e-04 | IGLV2-23 | 3.0 | 6.75e-05 | IGLV2-11 | 1.8 | 0.009 | IGLV1-44 | 3.6 | 0.002 | IGLV5-37 | -0.9 | 2.88e-04 |
| IGLV2-11 | 2.0 | 8.14e-04 | IGLV1-44 | 2.6 | 1.83e-04 | IGLV1-44 | 1.5 | 0.020 | IGLV1-47 | 2.8 | 0.002 | IGLV4-69 | 1.9 | 3.24e-04 |
| IGLV3-32 | -0.6 | 8.14e-04 | IGLV2-8 | 2.6 | 4.53e-04 | IGLV3-19 | 1.5 | 0.033 | IGLV2-8 | 2.9 | 0.003 | IGLV3-1 | 2.3 | 3.27e-04 |
| IGLV4-3 | -0.6 | 8.66e-04 | IGLV6-57 | 2.0 | 4.81e-04 | IGLV1-47 | 1.1 | 0.064 | IGLV3-10 | 2.2 | 0.007 | IGLV3-21 | 2.6 | 3.28e-04 |
| IGLV3-1 | 1.9 | 9.59e-04 | IGLV3-25 | 2.0 | 6.43e-04 | IGLV5-48 | -0.6 | 0.081 | IGLV3-1 | 2.7 | 0.007 | IGLV4-3 | -0.7 | 3.28e-04 |

| JO1 vs. NT | | | IMNM vs. NT | | | HMGCR vs. NT | | | SRP vs. NT | | | IBM vs. NT | | |
|---|---|---|---|---|---|---|---|---|---|---|---|---|---|---|
| Gene | LogFC | q-val | Gene | LogFC | q-val | Gene | LogFC | q-val | Gene | LogFC | q-val | Gene | LogFC | q-val |
| IGLV3-25 | 3.9 | 4.73e-11 | IGLV6-57 | 1.2 | 0.002 | IGLV6-57 | 1.0 | 0.019 | IGLV1-44 | 2.7 | 3.09e-05 | IGLV6-57 | 4.4 | 4.38e-18 |
| IGLV6-57 | 3.9 | 2.48e-10 | IGLV2-23 | 1.5 | 0.008 | IGLV5-52 | -1.3 | 0.028 | IGLV2-23 | 2.7 | 5.64e-05 | IGLV1-51 | 5.1 | 1.89e-13 |
| IGLV1-40 | 4.6 | 1.21e-08 | IGLV5-52 | -1.4 | 0.012 | IGLV5-48 | -0.5 | 0.040 | IGLV2-14 | 2.4 | 7.48e-04 | IGLV3-1 | 4.5 | 2.47e-13 |
| IGLV2-23 | 4.6 | 1.61e-08 | IGLV1-44 | 1.4 | 0.014 | IGLV2-23 | 1.1 | 0.066 | IGLV6-57 | 1.8 | 8.63e-04 | IGLV3-10 | 3.3 | 4.45e-13 |
| IGLV3-1 | 3.8 | 5.05e-08 | IGLV5-48 | -0.5 | 0.024 | IGLV1-44 | 1.0 | 0.107 | IGLV3-1 | 2.0 | 0.001 | IGLV2-23 | 6.0 | 8.21e-13 |
| IGLV1-44 | 4.3 | 5.55e-08 | IGLV1-40 | 1.1 | 0.031 | IGLV1-40 | 0.9 | 0.112 | IGLV2-11 | 2.0 | 0.002 | IGLV2-8 | 5.2 | 2.32e-12 |
| IGLV2-14 | 4.0 | 4.61e-07 | IGLV2-14 | 1.2 | 0.055 | IGLV3-32 | -0.4 | 0.112 | IGLV1-40 | 1.8 | 0.008 | IGLV1-44 | 5.1 | 2.54e-11 |
| IGLV2-8 | 4.1 | 4.62e-07 | IGLV5-37 | -0.5 | 0.056 | IGLV3-22 | -0.4 | 0.112 | IGLV2-8 | 1.9 | 0.008 | IGLV1-40 | 5.6 | 3.14e-11 |
| IGLV1-51 | 3.6 | 8.26e-07 | IGLV1-47 | 0.9 | 0.057 | IGLV1-47 | 0.8 | 0.121 | IGLV3-25 | 1.6 | 0.016 | IGLV2-18 | 2.8 | 5.25e-11 |
| IGLV2-11 | 3.8 | 1.16e-06 | IGLV3-32 | -0.4 | 0.059 | IGLV5-37 | -0.4 | 0.134 | IGLV3-21 | 1.4 | 0.039 | IGLV3-25 | 4.1 | 6.21e-11 |
